# 

**Published:** 1886-04

**Authors:** 


### BOOKS AND PAMPHLETS RECEIVED.

THE SKELETON IN GEOCOCCYX. By Dr. R. W. Shufeldt, U.S.A. Reprint from the *Journal of Anatomy and Physiology.* London: January, 1886.

TRANSACTIONS OF THE AMERICAN DERMATOLOGICAL ASSOCIATION. Ninth Annual Meeting held at Greenwich, Conn., August, 1885.

A NEW DEPARTURE IN UTERINE THERAPEUTICS; THE DRY TREATMENT. By George J. Engelmann, M.D. St. Louis.

NOTE BOOK FOR CASES OF OVARIAN AND OTHER ABDOMINAL TUMORS. Adapted from the note books of Sir Spencer Wells and the Samaritan Hospital, London, by John Homans, M.D. Published by Cupples, Upham & Co. Boston.

CONSTITUTION AND BY-LAWS OF THE VETERINARY MEDICAL ASSOCIATION OF NEW JERSEY. Paterson: 1885.

